# Whether intelligentization promotes regional industrial competitiveness: Evidence from China

**DOI:** 10.1371/journal.pone.0271186

**Published:** 2022-07-27

**Authors:** Bingjian Zhao, Yi Li, Junyin Tan, Chuanhao Wen

**Affiliations:** 1 Research Center for Economy of Upper Reaches of the Yangtze River, Chongqing Technology and Business University, Chongqing, China; 2 School of Economics, Xihua University, Chengdu, China; 3 School of Economics, Yunnan University, Kunming, China; University of Almería, SPAIN

## Abstract

Intelligentization-oriented development is a fast-developing trend of technological revolution. It promotes the reconstruction of the industrial system of a region and affects its overall industrial competitiveness. This paper sets up a variety of models featuring intelligentization level and multi-dimensional industrial competitiveness, and collects data of 28 provinces and cities in China from 2003 to 2017 to test the influence of industrial intelligentization level on the industrial competitiveness of a region. The result reveals that: 1) In China’s provincial jurisdictions, the higher the level of intelligentization is, the lower the overall level of industrial competitiveness and the lower the proportion of industry in the economic system will be. In regions where the facilities are highly intelligentialized, the production sectors tend to move to the less developed regions, and the growth effect of technological dividends is the focus. 2) Compared with the middle region and the Western region of China, the Eastern region, which is more developed with higher intelligentization level, has stronger ability in the research and development (R&D) of technologies, and the economic structure of the industry there tends to be stable, manifesting a strong growth potential.

## I. Introduction

Since 1978, China has seized the opportunity of the transfer of the global industrial chain and has greatly improved its industrial competitiveness [1, 2]. According to the global industrial competitiveness ranking released by the United Nations Industrial Development Organization, China rose from 61st in 1985 to 2nd in 2019 [3, 4]. At the same time, China is also facing the problem of imbalanced industrial development [5], especially the severe imbalance of the internal structure of the industry with a huge overall scale but under-developed technology [6]. Moreover, China’s industrial development is undergoing drastic changes as environmental problems in such pollution-intensive industries as steel, petrochemicals and building materials become increasingly prominent [7, 8].

Industrial competition is also competition among industries in essence, which is a dynamic process [9]. Manufacturers take actions on their relative competitive position, integrate their own resources by judging the market situation, reduce product prices, launch new products and other ways to gain market share and additional benefits [10, 11]. Regarding the acquisition of competitive advantage, absolute advantage theory proposes that the difference of the cost of production and efficiency leads to the difference of competitiveness [12]. Meanwhile, the comparative advantage theory holds that, an industry, even though having no absolute advantage, can still participate in international competition with certain comparative competitiveness [13], which was brought about by such resources endowment as production ability and innovation ability [14]. But in general, based on the perception of changes in the competitive environment, manufacturers must take actions to gain a competitive advantage [15]. After the Second World War, underdeveloped countries gradually realized the importance of industrial competition to fend off competition from developed countries [16]. Therefore, in the context of the Cold War, early industrial economists directly linked industrial competitiveness with national competitiveness, believing that national competitiveness depends on the competitive advantage of industries and enterprises, which in turn depends on the “national environment” [17]. When we turn our attention from a country to a region, industrial competitiveness is directly related to the competitiveness of the region, but the attention to the industry is more micro, involving mutual division of labor and association [18]. So when the level of regional development is inspected, industrial competitiveness is often the most important element, involving both such macro aspects as human capital, innovation, agglomeration and accessibility [19], and micro aspects as cost, structure and output [20]. Therefore, in today’s market economy system, the fierce market environment also requires the industrial sector to have a stronger survival ability, or competitiveness, and various factors affecting the industrial competitiveness have, consequently, become the focus of research [21–23]. In this process, technological upgrading plays a very important role, and research and development is the source of leadership [24].

Intelligent technology is the knowledge innovation carried out by human beings to solve and control complex systematic problems. When Intelligent technology is combined with various equipments in the production process, it will become “intelligentization”, such as artificial intelligence and industrial robots [25]. The rule-based expert system represented by Feigenbaum has been successfully developed and applied, providing powerful tools for industrial data analysis, metallurgical control, computer design, commercial and scientific applications, etc. This is the first industrial application of intelligent technology [26]. In the 1990s, with the breakthrough of technical limitations, intelligent industrial robots and service robots have been comprehensively developed and widely applied, resulting in the early prosperity of intelligent robot industry [27]. Around 2006, computer scientists Geoffrey Hinton, Yann Lecun, and Yoshua Bengio overcame the technical bottleneck of deep learning, marking a historic upsurge in the use of intelligent technology in production [28]. Today, intelligentization refers to the process of economic production, supported by High-speed Internet, Big data, Internet of Things, Artificial Intelligence and other technologies, industrial machines and computers imitating human behaviors or ways of thinking, so as to indirectly or directly replace human labor [29, 30]. The goal is to increase the efficiency of production and get better output [31].

In the information age, great changes have taken place in the industrial field. Intelligent technology is becoming a new driving force for industrial upgrading in various countries, helping production departments to cope with more difficult challenges [32]. In 2013, The United States released a number of strategic plans for artificial intelligence, and Germany started the era of “Industry 4.0”. In 2015 and 2017, China also formulated the “Made in China 2025” and the “Next Generation AI Development Plan” [33]. A collation of the latest research shows that Artificial intelligence and the Internet, as general technologies, have considerably promoted complementary innovation and helped enterprises improve productivity by supplementing or replacing human resources, so as to complete more complex tasks, thus bringing about multiplier effect [34–36]. At present, intelligent technology is deeply integrated with industry, and it will be more common for intelligent products to replace labor force,and enterprises are also willing to improve productivity by replacing labor force with advanced technological capital [37–40]. The level of intelligentization has become an important factor affecting the competitiveness of enterprises, and it is more and more common for industry and manufacturing industry to have intelligent technology [41–43]. From the perspective of economy, the application of intelligent technology can, first of all, effectively improve the product innovation ability of enterprises, and then quickly promote industrial upgrading, enabling enterprises to gain advantages in the industry and global competition and in the end achieve revenue growth with a more qualified, efficient, profitable and flexible structure [44, 45]. With respect to the environment, industrial competitiveness must take the factor of environment into consideration [46]. Intelligentization will reduce the output of pollution in economic development and bring about a positive impact on the environment [47]. Therefore, it is confirmed that intelligent technology will equip the industrial development with sustainable competitive advantages [48, 49].

**Fig 1:**
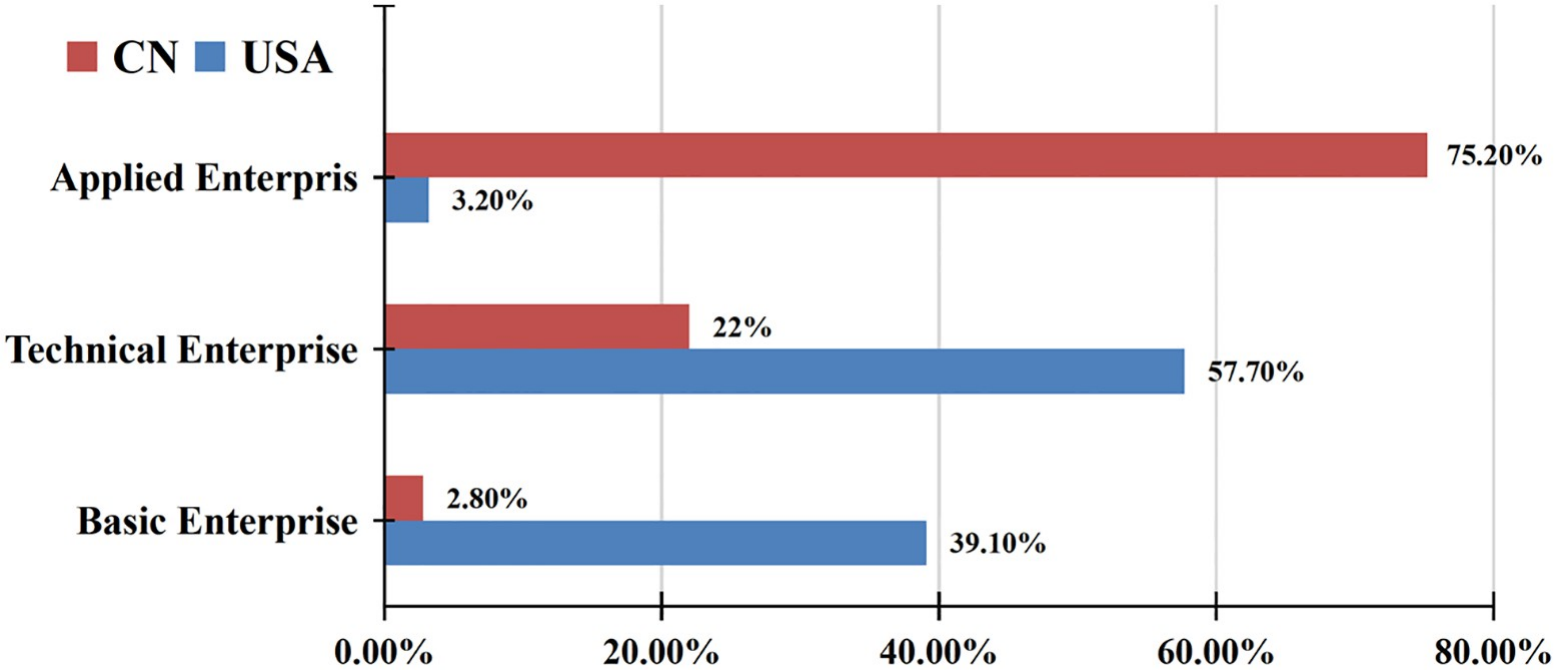
Distribution of AI enterprises in China and the United States. (Data source: China’s New Generation AI Technology Industry Development Report (2019), Evergrande Research Institute).

**Fig 2:**
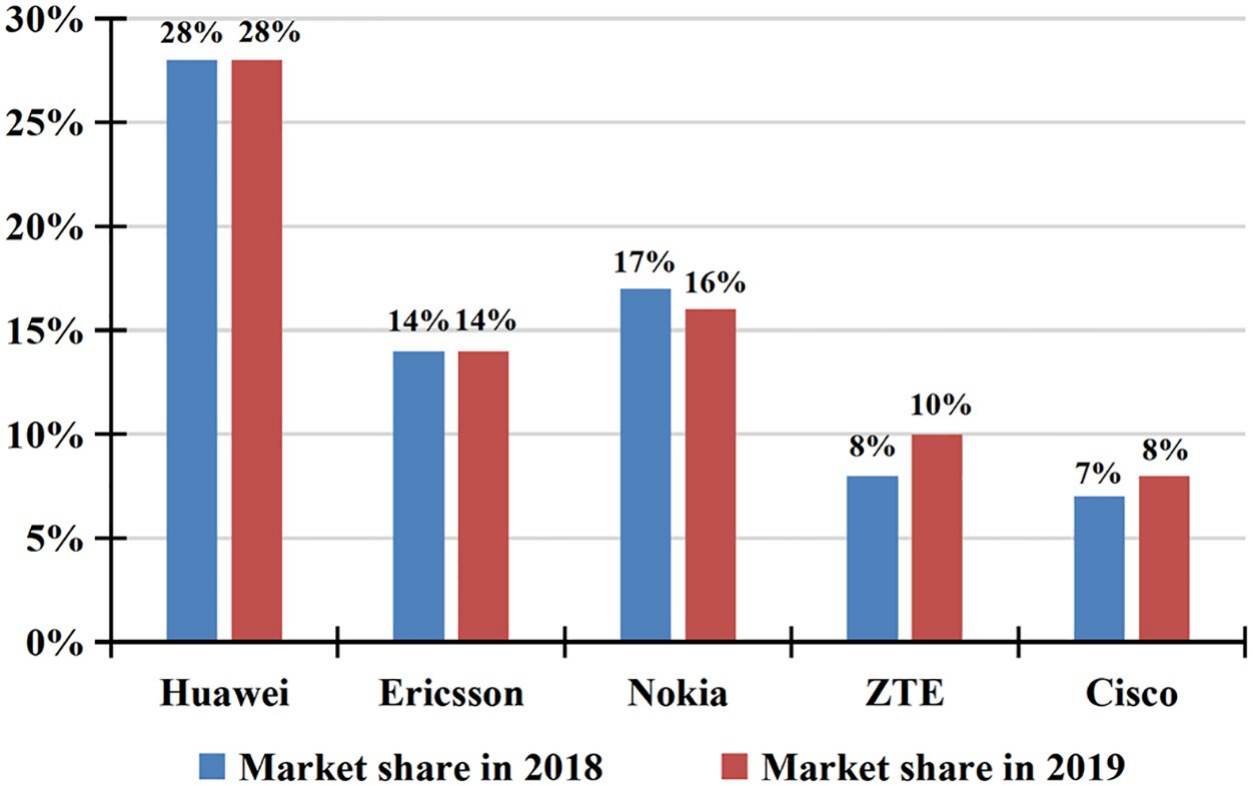
Global telecom equipment market revenue 2018–2019. (Data source: China’s New Generation AI Technology Industry Development Report (2019), Evergrande Research Institute).

As mentioned above, China is developing ambitious plans for industrial intelligentization and is leading the global development of intelligent technology, in which industrial intelligence upgrading is the core of China’s development [50–52]. In contemporary society, in terms of technology, China’s exploration of intelligent technology has formed a unique and rich system, with its own technological advantages in information processing, machine learning, industrial Internet and e-commerce [53]. However, compared with the United States, China pays more attention to the application of intelligent technology, with as high as 75.2% of Chinese enterprises employing AI (Fig 1), which enables the technology to contribute to profits in a quick fashion. In electronics, the core of intelligent technology, Chinese enterprises take on prominent advantage in the world’s major manufacturers, if the impact of trade war with U.S.A. is factored out (Fig 2) [54, 55]. Now, a complete intelligent technology system and industrial chain have been established in China, and intelligentization has become the inevitable trend of industrial upgrading [56, 57]. The Chinese Government attaches great importance to the formulation of relevant policies and is committed to industrial intelligentization upgrading [58, 59]. In the Chinese industrial sector, the number of industrial robots has maintained a rapid growth with an average annual growth rate of around 20–30%. According to the large data, the industrial output value will reach $15 billion over the next five years, while the application value of AI will reach $20 billion (National Bureau of Statistics of China, 2019). Predictably, China is likely to be the world leader in intellectual technology and related high-tech industries.

**Fig 3:**
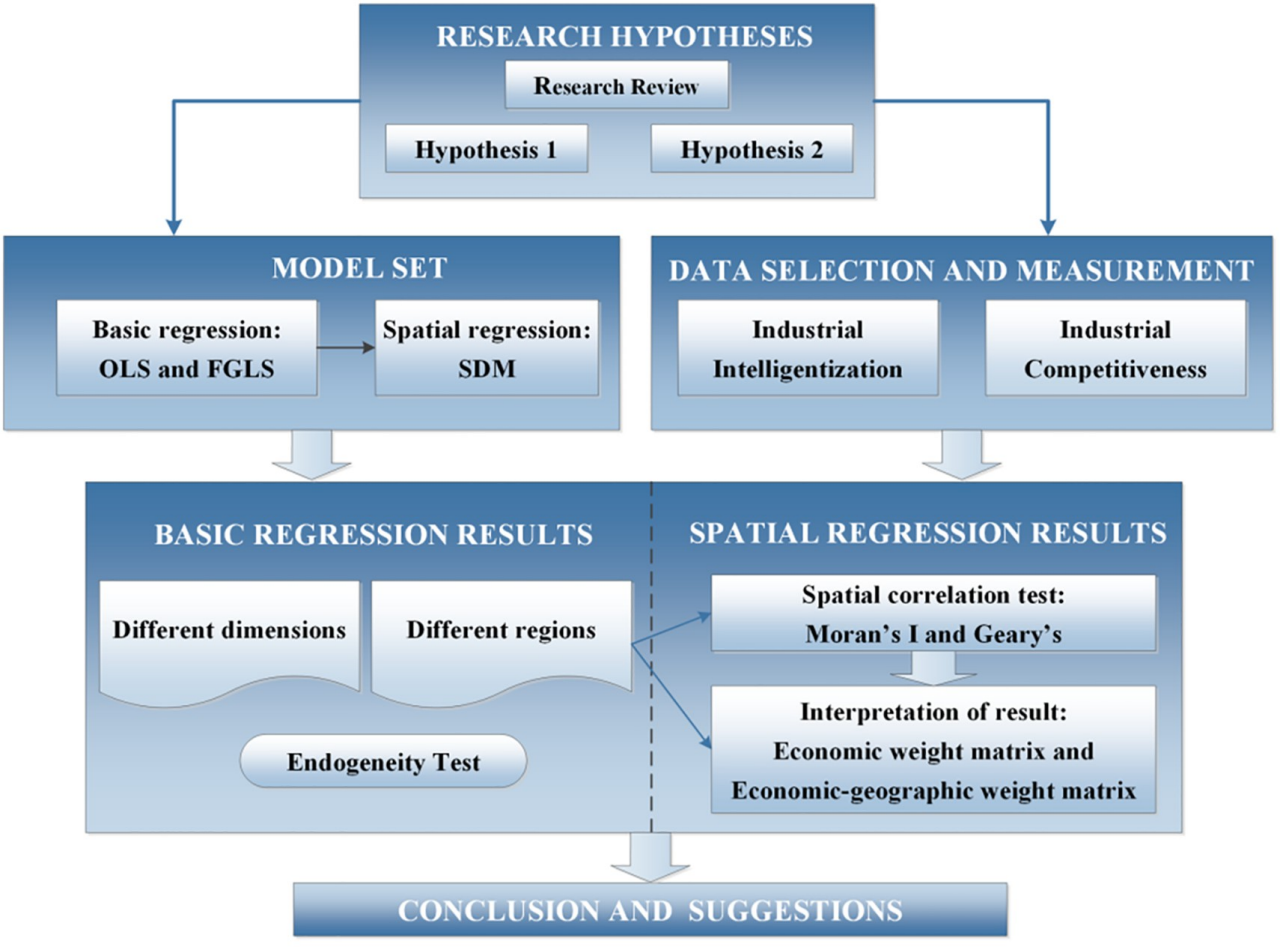
Framework diagram of this paper.

For developing countries, how to influence industrial development through intelligentization also needs a successful case. China has the most complete industrial system and the fastest developing intelligent technology system in the world. At present, industrial sectors in different regions of China are experiencing the impact of intelligent technology, and the differences between developed regions and undeveloped regions are very obvious. So, it is of vital significance to study the relationship between the industry intelligentization and Chinese industrial development so as to help promote the industrial competitiveness of emerging developing countries [60, 61], which at the same time help to improve and supplement the industrial theory in the intelligent era [62]. Based on the above reasons, this paper designs a targeted research framework to analyze the relationship between intelligentization and industrial competitiveness of various regions in China (Fig 3). Compared with the existing literature, the innovation of this study lies in three aspects: (1) Currently, the study on intelligentization is mainly about the successful experience of developed countries, such as Germany’s Industry 4.0. This paper focuses on China, a developing country with the fastest development of intelligent technology in the world, to discuss the relationship between intelligentization and industrial competitiveness in developing countries. (2) At present, the research on intelligent technology mainly focuses on the micro level, for instance the change of the internal structure of the industry, which belongs to the category of industrial economics. The research perspective of this paper is macro level, taking regions as the research object, focusing on the change of industrial competitiveness between regions in the era of intelligentization, which belongs to the category of economic geography. (3) According to the actual situation of the development of intelligent technology in China, this paper improves and constructs the index system to evaluate the level of intelligentization. At the same time, the ecological niche theory is introduced for the first time in the process of evaluating industrial competitiveness of each region.

## II. Research hypotheses

This paper takes China’s provincial jurisdictions as the research object, and reviews the process of industrial development from the perspective of regional economics. The most direct impact of the changes of external factors on industries and industrial sectors is usually the transfer or flow of industrial elements and relevant knowledge, which brings about the change of location of industry, the impact of which on the local economic development can never be overlooked [63]. In contrast, when an industrial sector is looking for an ideal location, the relationship among factors affecting economic activities should be taken into consideration [64]. The same is true to industry. The industrial location theory proposed by Weber is an important theoretical basis for location selection, which comprehensively introduce the influencing factors of industrial location [65]. For example, factors as trade, agglomeration and infrastructure will affect the location selection of industrial sectors [66, 67]. Because industrial location is closely related to competitiveness, changes in location also indicate changes in the competitiveness of industrial sectors [68]. Therefore, this paper will discuss how intelligentization would affect industrial competitiveness from the perspective of industrial transfer.

In 1961, Posner, an American technology and trade economist, put forward the “technology gap Theory”, pointing out that the technology gap is the main factor for the inter-regional flow of products in the industrial era [69]. Based on the “technology gap Theory”, Vernon proposed the product life cycle theory. According to Vernon, every product has its own life cycle, going through birth, growth, maturity and decay. The most important idea of the theory is that when a product reaches maturity, the production process becomes standardized and widely accepted, so that there is no need for many skilled workers to continue to produce the product. In order to save costs, the developed region that originally produced the product will transfer the production process to the less developed region [70]. According to these theories, the most developed regions are always keen on non-standardised products that contain new and more advanced technologies [71]. In the 1980s, Gort, Klepper and other scholars developed the product life cycle theory into the field of industry, forming the “Industry life cycle theory” [72, 73]. As for how to overcome the downturn of the industry, there are two main focus fronts at present: one is the internal operation of the industry, the other is the external location of the industry. Within the industry, it is generally the innovation activities that enhance the enterprise vitality to delay the industrial recession. In the choice of industrial location, regional transfer is advocated to maintain industrial efficiency [74].

When the impact of innovation and location on industry is taken into account, the main point of this paper can be extended, that is, the industrial gradient transfer caused by technological innovation, which is the development of industry life cycle theory in the field of economic geography [75]. As the life cycle of an industry changes, the location of production will eventually change, leading to industrial transfer [76]. The Japanese economist Kaname Akamatsu proposed in the 1930s the “flying geese pattern” theory of industrial development, that is, economically-developed regions play the leading goose, the developing regions fly in the middle, and the under-developed areas take the tail position. As the leader, the developed areas actively develop the knowledge and technology-intensive industries and at the same time pass to the developing and under-developed areas the ripen industries through trade, technology transfer and direct investment, thus forming a “flying geese pattern” [77]. Kojima further argued that industrial transfer should basically follow the order of industrial evolution, that is, the transfer should be carried out in the order of resource-intensive, labor-intensive, capital-intensive and technology-intensive [78]. Specifically, with the improvement of the level of industrialization and the maturity of industrial sectors, labor costs will continue to rise, and labor-intensive industries will gradually lose their comparative advantages. Developed regions will transfer labor-intensive industries to underdeveloped regions, while strengthening the development of technology-intensive industries to maintain their technological advantages [79].

China’s industrial development is extremely mature, and it has formed a complete standardization system. From the perspective of industrial life cycle, the industrial systems of China’s developed provinces have entered a mature area, even a period of decline [80, 81]. From the perspective of technological innovation, developed regions will further develop technology-intensive industries by virtue of the advantages of intelligent technology. Does this mean that less developed areas will become centres of industrial development? Will the advantageous area of traditional industry shift?

**Hypothesis 1:** According to the “industrial gradient shift theory” of location choice, intelligent technology as a new impact factor, will promote the industrial sector to shift from developed regions to the undeveloped areas, and the latter will gain stronger industrial competitiveness.

Each industry is composed of different sectors, and its competitiveness is also manifested in market influence, industrial growth, resource allocation, structural transformation, industrial innovation and other aspects [82]. According to the “flying Geese model” theory, labor-intensive, resource-intensive and capital-intensive industries will first move to developing regions. Economically developed areas generally have advantages in technology and capital, but not in labor cost. So on the one hand, some capital which has no advantage or external economic activities will transfer from the economically-developed regions to the less developed regions; while on the other hand, some factor endowments will outflow from the under-developed regions to the developed regions. But more importantly, the new capital will be mainly allocated in the developed areas. For instance, in a certain period of time, enterprises with low production efficiency will be closed, but at the same time, more and more advanced enterprises will be established [83, 84]. The core of intelligentization is intelligent technology, which is an intermediate product in the industrial chain. On the one hand, in order to prevent other intermediate product manufacturers from obtaining monopoly profits through continuous innovation of intermediate products, R&D departments will compete to increase R&D investment to improve the production efficiency and production technology level of intermediate products. On the other hand, the final product itself will in turn affect the efficiency of innovation and generate new endogenous driving force [85, 86]. In the Eastern part of China, the so called “marginal industries” which are resource-intensive or resource-dependent, still account for a large proportion and are huge consumers of energy and raw material. With the drying-up of natural resources and the rising price of raw material in the East, many above mentioned enterprises are facing the great pressure of survival and development. Consequently, the Eastern region will transfer the production sector to the West, either out of the need for adjusting the industrial structure or the retention of cost advantage, meanwhile it will enhance the competitiveness of R&D by further drawing on the technological advantages so as to obtain the growth advantage. In general, in the era of continuous innovation of intelligent technology, developed regions will further shift the industrial focus from production to R&D, so as to obtain stronger competitiveness in technological innovation.

**Hypothesis 2:** The developed region does not transfer the entire industrial system to the undeveloped region, but transfers the production department out, and retains the research and development department itself, which exists the phenomenon of “relative transfer”.

## III. Model and methodology

### 3.1 Model set

In light of the above analyses and hypotheses, the main purpose of this paper is to analyze the effect of intelligentization on industrial competitiveness. With the development of mathematical statistics, metrology is often used to discuss the influence of one factor on another in social science, psychology, medicine and other fields [87, 88]. According to the metrological methodology, we first need to understand the fundamental impact of intelligentization on industrial competitiveness in each region [89, 90]. The development of intelligent technology belongs to technological progress, so the econometric model can be constructed from the perspective of technological progress to economic development [91]. Like a large number of studies, we first constructed the following “linear regression model” as a benchmark [92–94]:

$$Comp_{ijt}=\alpha_{0}+\alpha_{1}Int_{it}+\alpha_{2}X_{it}+\mu_{i}+\varepsilon_{it}$$ (1)

In this model, *Comp*_*iqt*_ represents the competitiveness level of *j* dimension of *i* region in period; *Int*_*it*_ represents the level of industrial intelligentization of *j* dimension of *i* region in *t* period; the unobservable random variable *μ*_*i*_ is the intercept representing individual heterogeneity, *ε*_*it*_ is the disturbance term that changes with individual and time, *α*_0_ is the constant intercept term, *α*_1_ and *α*_*c*_ are the related coefficients (the same below), and *X*_*it*_ is the control variable.

The economic system is a complex and giant system composed of multiple elements, and the interrelation between variables is often caused by spatial relations. Eq (1) only investigates the impact trend of intelligentization on the change of regional industrial competitiveness in relation to time, but it does not take into consideration factors such as spatial dependence, which may thus lead to biased regression results [95]. In the region involved in this research, the spatial factor is the factor that needs to be investigated [96, 97]. Therefore, in order to figure out whether intelligentization level has spatial independence in the regions, this paper introduces spatial factors into the traditional regression model, which entails that “spatial econometric model” needs to be selected [98]. The “Spatial Dubin model” is a form of spatial econometric model. Compared with basic regression model (1), it assumes that the explained variables of one region are affected by the explanatory variables of other regions [99]. In this paper, the competitiveness transfer of the industry is shown as the center of the industry changes from one region to another, so the following spatial Dubin model needs to be constructed.

$$Comp_{it}=\beta_{1}Int_{it}+\beta_{2}\sum_{j=1}^{n}W_{ij}Comp_{jt}+\beta_{3}\sum_{j=1}^{n}W_{ij}Int_{jt}+\beta_{4}X_{it}+\varepsilon_{it}$$ (2)

Wherein, *W*_*ij*_ is the spatial weight matrix of row *i* and column *j* (*i* = 1,2,3…n; *j* = 1,2,3…n). $\sum_{j=1}^{n}W_{ij}Comp_{jt}$ represents the spatial lag item of the explained variable, $\sum_{j=1}^{n}W_{ij}Int_{jt}$ represents the spatial lag of the core explanatory variable, *X*_*it*_ is the control variable, *ε*_*it*_ is the perturbation term that changes with individual and time.

According to the assumption of the classical model of spatial economics, there are three main types of spatial weight matrix: Geographical Matrix (*W*_1_), Economic Matrix (*W*_2_), Economic-Geographic Matrix (*W*_3_) [100, 101]. Considering that intelligent technology is the further development of Internet and information technology, and it has broken through the limit of distance, this paper adopts the Economic Matrix(*W*_2_) and Economic-Geographic Matrix(*W*_3_). Different spatial weight matrices can reflect the spatial correlation between different provinces, their expressions are as follows [102, 103]:

- Eeographical Matrix(*W*_1_): 1d2(i≠j)0(i=j)
- Economic Matrix(*W*_2_): 1Gpi−Gpj(i≠j)0(i=j)
- Economic-Geographic Matrix(*W*_3_): W3=12W1+12W2

Wherein, *d* is the distance between the two regions, $G_{p_{i}}$, $G_{p_{j}}$ is the GDP of the *i* or *j* province during the study period.

### 3.2 Data selection and measurement

#### 3.2.1 Core explanatory variable-level of industrial intelligentization(*Int*)

Currently, there are not many representative index systems to measure the level of intelligentization, and even fewer literature to measure the level of industrial intelligentization. The relevant index system mainly measures the level of informatization or internetization, so the core explanatory variable system in this paper is not limited to the research of some authoritative scholars [104, 105]. At the same time, some actual situation of China should be taken into consideration and the index systems raised by some representative Chinese scholars borrowed [106, 107]. The whole index system mainly includes the following three dimensions (Table 1). Factor Analysis is adopted to measure the industrial intelligentization level of each region (For processing convenience, 1 is added to the standardized comprehensive score obtained).

**Table 1:** Evaluation index system of intelligentization level.

Industrial Intelligentization Level	First Level Index	Second Level Index	Index Property	Explanation
	Investment in intelligent equipment	Popularity of related software	positive	The proportion of application software, embedded application software, industrial software and other software products to the main business revenue of all industrial enterprises
		Intelligent equipment application	positive	The proportion of imports of computers, electronic components, instruments and equipment to the main business income of all industrial enterprises
		Information resource collection and data processing capability	positive	The proportion of system integration and service revenue, information consulting and management service revenue, software service revenue in the main business revenue of all industrial enterprises
	Production capacity of high-tech enterprises	Profits from high-tech industries	positive	The proportion of the main business income of high-tech industries in each province to the national main business income of high-tech industries
		New product production	positive	The proportion of new product sales revenue in the main business income of industrial enterprises
	Internet Infrastructure	Accessibility of the working-age population to the Internet	positive	The percentage of people online in the population aged 15–64
		Construction level of Internet access equipment	positive	Number of Internet ports per 10,000 people
		Construction of fiber optic infrastructure	positive	Length of long distance optical cable lines per square kilometer

#### 3.2.2 Core explained variable-industrial competitiveness(*Comp*_*it*_)

As for the measure of industrial competitiveness, this paper defines and measures it on the theory of niche situation. Ecological niche in ecology is the study of the relationship among time, space, and function and relative status. Specifically, it refers to the relative position, level of a species in time and space resources and external environment and the functional relationship with the other related species.

Scholars as Dimmick [108], Baum&Olive [109] have introduced the idea of ecological niche into the study of industrial competitiveness. The ecological niche defined in this paper refers to the relative position and functional position of the resources occupied by industry in a certain region in the regional industrial ecosystem and the relationship between them and production services in other regions. The biggest difference between the niche in this paper and the biological niche is this: due to the significant initiativeness of economic industry, industries of a region will voluntarily choose to join in or withdraw from the competition. Accordingly, this paper adopts the Niche “Ecostate-Ecorole” model (3) proposed by Zhu [110, 111], which divides industry development into its current state and its future potential. The current state refers to the existing state and competitive advantage of the industry based on its own growth and development, as well as the interaction between the industry and the environment, which reflects the survival capacity. In this paper, the current state level is represented by relevant index data of each year from 2003 to 2017. The future potential of an industry refers to the trend and potential of its development and change, a portrait of the dominance and influence of certain environment, thus reflecting the capacity of development of the industry. In this paper, the potential value is represented by the annual average growth value of the relevant index data from 2003 to 2017. By comprehensive calculation of the current state and future potential of the industry-related indicators, the niche width, or the size of the industrial niche can be obtained, which reflects the strength of the industrial competitiveness of a region or the relative position in the regional industrial system.

$$N_{i}=\left(S_{i}+A_{i}P_{i}\right)/\sum_{j=1}^{n}\left(S_{j}+A_{j}P_{j}\right)$$ (3)

Wherein, *N*_*i*_ refers to the niche of industrial competitiveness index *i*, *S*_*i*_ is the current state of industrial competitiveness index *i*, *P*_*i*_ is the potential of industrial competitiveness index *i*, *S*_*i*_+A_*i*_P_*i*_ represents the absolute niche of productive industrial competitiveness index *i*. A_*i*_ represents the dimensional conversion coefficient, and la is used as the dimensional coefficient (1a is the time scale. Since the time scale is in the unit of year, the dimensional conversion coefficient in this paper adopts "1"). The ecological niche or comprehensive ecological niche being jointly determined by multiple index factors, the calculation formula of the industrial comprehensive ecological niche in a certain area is shown in the following model (4).

$$M_{i}=\sum_{i=1}^{n}N_{i}W_{i}$$ (4)

Wherein, *M*_*i*_ represents the comprehensive ecological niche of the industry or the ecological niche of a certain dimension, and *W*_*i*_ represents the weight corresponding to the index *i*. Many scholars in biology argue that multiple dimensions of ecological niche act together, in other words, the subject species itself faces a multi-dimensional environment [112–114]. Drawing on this concept, holding that industrial competition also happens in a multi-dimensional situation, this article determines the four competitiveness dimensions: the Basic Dimensions (*Bas*), the Structure Dimension (*Cons*), the Potential dimension (*Pote*), and the Innovation Dimension *(Inno*). The basic competitive dimension represents basic industrial production capacity, and is mainly related to production sector; the structure dimension represents the proportion of industry in the whole economic system; the potential dimension indicates the industry’s growth potential, and the innovation dimension stands for technical innovation ability and is related to R&D sector. In addition, This paper assumes that less inventory means higher social recognition and shorter sales cycle. The application of intelligent technology will directly affect the micro financial ability of enterprises. Financial level plays a core role in creating value and reducing cost of enterprises. It can significantly improve the efficiency of capital operation of enterprises, thus enhancing the macro-operation level of enterprises and improving their competitiveness. Based on this consideration, this paper adopts the micro financial benefit index in the intelligent index, and regards the asset operation and total income level as the macro competitiveness, so as to reflect the difference between the operation process and the operating income, as is shown in Table 2:

**Table 2:** Multidimensional niche evaluation index system of industrial competitiveness.

First Level Index	Second Level Index	Third Level Index	Index properties	Explanation
Level of Industrial Competitiveness	The Basic Dimensions	Total Asset’s Contribution Rate	positive	(Total Profits+Total Taxes+Interest Expense) / Average Total Assets
		Ratio of Profits to Cost	positive	Total profit/Total cost
		Current Assets Turnover	positive	Net Income From Main Business / Average Total Current Assets
		Sales of Industrial Products	negative	Industrial Stock above Designated Size / Industrial Capital Stock
		Total Income Level	positive	Total Revenue from Main Business Above Designated Size / Total Assets above Designated Size
	The Structure Dimension	The Proportion of Industrial Output	positive	Industrial Added Value / Gross GDP
		Employment Ratio	positive	Number of People Employed in Industry / Number of People Employed in the Whole Society
		The proportion of tax	positive	Total Industrial Revenue / Total National Revenue
		The Ratio of Investment in fixed Assets	positive	Industrial Fixed Asset Investment / National Fixed Asset Investment (Excluding Rural Households)
	The Potential Dimension	Employment Trend	positive	The Growth Rate of Industrial Employment
		Fixed Investment Trend	positive	The Growth Rate of Industrial Fixed Assets Investment (Excluding Rural Households)
		Tax Trend	positive	The Growth Rate of Industrial Tax Collection
		Trend of Output Value	positive	The Growth Rate of Industrial Added Value
	The Innovation Dimension	R&D Expenditure	positive	Internal Expenditure of Industrial Enterprises’ R&D Funds/Internal Expenditure of R&D Funds
		R&D Project	positive	Average Number of R&D Projects / Number of Industrial Enterprises
		Scale of R&D Personnel of The Enterprise	positive	Average Annual Number of R&D Personnel in Industrial Enterprises / Employees in Industrial Enterprises Above Designated Size
		Patent Output	positive	Number of Patent Applications for Industrial Enterprises / Full Time Equivalent of R&D Personnel

#### 3.2.3 Control variable

In econometric models, not only the influence of core variables on the explained variables, but also the influence of other factors must be considered to ensure the accuracy of the results. In this paper, we refer to relevant literature on influencing factors of industrial competition, and select education level (*Ec*), trade level (*Tra*), government education expenditure (*Es*), capital stock (*Hc*) and urbanization level (*Ur*) as control variables [115–119]. Table 3 shows the specific contents of control variables.

**Table 3:** The index system of control variables.

Indicators	Index Property	Explanation
Education level	positive	The average number of years of education of the population over 6 years old
Trade level	positive	The proportion of total imports and exports in GDP of each region
Government education expenditure	positive	The proportion of education expenditure in fiscal expenditure
Capital stock	positive	The capital stock per capita
Urbanization level	positive	The proportion of urban population in the total population

#### 3.2.4 Data analysis

China’s industrial classification has been revised several times, and the most recent second revision is in 2002, on which most relevant industrial studies are based. Therefore, this paper takes 2003 as the base period and uses data from 2003 to 2017. As there is large missing data of Qinghai, Tibet and Xinjiang provinces and interpolation would produce distortion, the paper uses the rest 28 provincial jurisdictions in China as the research sample. Data sources include *China Electronic Information Industry Statistical Yearbook*, *China Statistical Yearbook*, *China Industrial Statistical Yearbook*, as well as local statistical yearbooks of provincial jurisdictions in China, EPS Database and CSMAR Database. The descriptive statistical analysis of relevant variables in this paper is shown in Table 4. Meanwhile, Table 5 presents Pearson correlation among variables. According to the results, explanatory variables, control variables and explained variables are significantly correlated, indicating that there may be functional relationships among variables.

**Table 4:** Descriptions of the main variables.

Type of variable	Variables	Sample Size	Average	Standard Deviation	Minimum	Maximum
*Explained variable*	*Comp*	420	6. 252	0. 001	6. 250	6. 254
*Explanatory variables*	*Bas*	420	1. 501	0. 054	1. 407	1. 592
	*Cons*	420	1. 656	0. 048	1. 538	1. 733
	*Pote*	420	1. 603	0. 100	1. 439	1. 785
	*Inno*	420	1. 492	0. 054	1. 342	1. 591
	*Int*	420	1. 000	0. 751	1. 159	5. 556
*Control variables*	*Ec*	420	8.686	1.015	6.040	12.502
	*Tra*	420	0.462	0.883	0.032	7.001
	*Es*	420	0.163	0.025	0.111	0.222
	*Hc*	420	1.722	0.174	1.303	2.342
	*Ur*	420	0.518	0.147	0.248	0.896

**Table 5:** Correlation between variables.

	*Int*	*Bas*	*Cons*	*Pote*	*Inno*	*Comp*	*Ec*	*Tra*	*Es*	*Hc*	*Ur*
*Int*	1.000										
*Bas*	-0.717	1.000									
*Cons*	-0.079	0.085	1.000								
*Pote*	0.290	-0.799	-0.437	1.000							
*Inno*	0.251	0.409	-0.176	-0.669	1.000						
*Comp*	0.195	-0.429	-0.200	0.424	-0.166	1.000					
*Ec*	0.951	-0.689	0.116	0.189	0.237	0.252	1.000				
*Tra*	0.472	-0.408	-0.027	0.086	0.273	0.023	0.504	1.000			
*Es*	0.307	-0.358	0.196	0.083	0.033	0.389	0.406	0.268	1.000		
*Hc*	0.951	-0.755	0.086	0.279	0.163	0.336	0.974	0.509	0.392	1.000	
*Ur*	0.966	-0.672	-0.044	0.216	0.315	0.287	0.958	0.484	0.413	0.9489	1.000

## IV. Results and analysis

### 4.1 Basic regression results

Regression is performed on Eq (1). Firstly, OLS regression is used in this paper, and it is more reasonable to select fixed effect through Hausman test, In this paper, both time and region are fixed. Due to the adoption of panel data, further tests are required between regressions. First, there is no obvious between-group heteroscedasticity in OLS regression results. However, the correlation test mentioned above only proved the correlation between cross-sectional data, so the further test in this paper found that there was a correlation between inter-group autocorrelation and intragroup autocorrelation among variables, which had a great impact on the results. Therefore, this paper adjusts the estimation by iterative FGLS. The estimated results are shown in Table 6. Through the comparison of the results, it is indicated that the adjusted estimates promote little change in the overall trend. Therefore, the results are considered valid and robust. The following analysis is also based on the results of FGLS.

**Table 6:** Influence of intelligentization on industrial competitiveness.

Variables	*Comp*-(1)		*Bas*-(2)		*Cons*-(3)		*Pote*-(4)		*Inno*-(5)	
	OLS	FGLS	OLS	FGLS	OLS	FGLS	OLS	FGLS	OLS	FGLS
*Int*	-0. 001** (-2. 31)	-0. 001*** (-21. 62)	-0. 015 (-1. 48)	-0. 010*** (-8. 89)	-0. 038*** (-41. 4)	-0. 039*** (-55. 75)	0. 055** (2. 73)	0. 026*** (18. 62)	-0. 003 (-0. 25)	0. 006*** (6. 27)
*Ec*	-0. 001**(-3. 61)	-0. 001*** (-19. 64)	0. 033*** (3. 37)	0. 010*** (6. 45)	0. 063*** (6. 32)	0. 045*** (47. 71)	-0. 120*** (-6. 52)	-0. 022*** (-13. 19)	0. 024* (1. 92)	0. 007*** (5. 00)
*Tra*	-0. 0001 (0. 89)	-0. 0001*** (-14. 14)	-0. 001 (-0. 30)	-0. 002*** (-10. 54)	-0. 001 (-0. 92)	-0. 001*** (-6. 54)	-0. 002(-0. 34)	0. 003*** (14. 09)	0. 005 (0. 97)	0. 002*** (14. 80)
*Es*	0. 016*** (4. 43)	0. 014*** (26. 14)	-0. 380*** (-2. 85)	-0. 207*** (-12. 94)	0. 530*** (3. 97)	0. 646*** (48. 10)	0. 256 (0. 99)	-0. 355*** (-38. 36)	-0. 389*** (-2. 87)	-0. 283*** (-19. 42)
*Hc*	0. 008** (5. 23)	0. 007*** (25. 43)	-0. 417*** (-10. 74)	-0. 321*** (-22. 69)	0. 170*** (3. 46)	0. 215*** (43. 90)	0. 707*** (9. 82)	0. 449*** (31. 10)	-0. 452*** (-9. 16)	-0. 395*** (-33. 71)
*Ur*	-0. 001 (-0. 69)	-0. 001*** (-4. 74)	0. 028(-1. 31)	0. 016 (1. 52)	-0. 316*** (-3. 78)	-0. 305*** (-35. 69)	0. 092*** (0. 47)	0. 052*** (-8. 50)	0. 195** (2. 26)	0. 165*** (9. 02)
*Constant*	6. 244*** (4350. 75)	6. 245*** (17400)	2. 015*** (31. 91)	2. 115*** (0. 018)	0. 965** (-2. 21)	1. 049*** (52. 08)	1. 306*** (8. 65)	0. 932*** (-0. 75)	1. 958*** (25. 20)	2. 042*** (-6. 10)
*Fixed effect*	Yes	Yes	Yes	Yes	Yes	Yes	Yes	Yes	Yes	Yes
*R* ^*2*^	0. 2041	-	0. 5935	-	0. 1743	-	0. 1245	-	0. 1897	-
*F*	45. 06***	-	171. 29***	-	59. 70***	-	36. 30***	-	59. 43***	-
*Wald*		12270***	-	6563. 63***		10236. 29***		10644. 03***		2278. 82***
*Hausman*	31.27***		36.75***		51.91***		11.18*		23.48***	
*Greene Test*	15. 62	-	2. 97	-	4. 95	-	1. 41	-	2. 56	-
*Wooldridge Test*	247. 801***	-	59. 800***	-	38. 934***	-	4. 689**	-	79. 983***	-
*Frees Tset*	18. 431	-	19. 228	-	19. 188	-	22. 004	-	21. 572	-

Note:

***, **, * represent the significance at the level of 1%, 5%, and 10% respectively. The data in parentheses are either t values or z values. (The same below)

Models (1)-(5) test the overall competitiveness level and the competitiveness level of each dimension respectively. Result of model (1) shows that the coefficient of intelligentization level is significantly negative, indicating that with the increase of intelligentization level, the overall industrial competitiveness level of a region is declining. In the results of model (2) and model (3), the coefficient of intelligentization level is significantly negative. Since the basic competitiveness represents the most basic production and operation capacity, the negative coefficient indicates that the region with a higher level of intelligentization takes on a declining competitiveness of industrial production, and the same is true for the proportion of industrial economy in the whole economic structure, which reflects the crowd-out effect of intelligent technology on industry and the fact that the higher the intelligentization level, the lower the industrial production capacity. However, in the results of model (4) and model (5), the coefficients of the core explanatory variable are significantly positive, which indicates that higher level of intelligentization means higher level of innovation and R&D, and stronger industrial growth capacity and greater potential of the whole region. The above findings show that on the one hand, the regions with a higher level of intelligentization have a smaller scale of industry in its economic aggregate, which reflects the trend of de-industrialization in the industrial centers in the technologically developed regions and the trend of production center shifting from the economically and technologically developed regions to the undeveloped regions—the reason for the fact that the lower intelligentization, the higher competitiveness level of industrial production. On the other hand, as further development of internet and information technology, intelligent technology has brought about enormous R&D dividends. The economically developed regions will make more investment in R&D, thus compared with the developing regions, they will demonstrate stronger innovation competitiveness. The technology dividend also leads to stronger economic growth momentum. Therefore, hypothesis 1 is verified that China’s industrial center is shifting from the developed regions to undeveloped regions. Meanwhile, it has also partly verified hypothesis 2, that there is a regional heterogeneity in the industrial transfer, which means that industrial transfer does not exist in all industrial sectors.

To ensure the robustness of the results, the provincial jurisdictions studied in this paper are further divided into Eastern, Central and Western China according to the convention of regional division in China. In particular, Eastern China included Beijing, Tianjin, Hebei, Liaoning, Shanghai, Jiangsu, Zhejiang, Fujian, Shandong, Guangdong, Hainan; Central China included Shanxi, Inner Mongolia, Jilin, Heilongjiang, Anhui, Jiangxi, Henan, Hubei, Hunan; Western China included Guangxi, Chongqing, Sichuan, Guizhou, Yunnan, Shaanxi, Gansu, Ningxia. Table 7 reports the regression results of the impact of multi-dimensional intelligentization-oriented development in each part on industrial competitiveness. The results of model (6)-(8) show the outcomes of the basic competitiveness dimension, which indicate that all the coefficients of explanatory variables are significantly negative, consistent with the previous results. Moreover, there is an obvious trend of regional extension. In other words, the further the inland, the greater the absolute value of the coefficient, indicating that the transfer of production sectors is stronger in Western China than in Central China and Eastern China. Results of model (9)-(10) exhibit the outcomes of the structural competitiveness dimension. The coefficients of all parts are also significantly negative, and the absolute value of the coefficients of Western China is greater than that of Central and Eastern China. The results reveal that the city with a higher level of intelligentization-oriented development in the Western China suffers faster decline of the proportion of industry in the economic structure, while the lowest decline degree lies in the Central China. The results of model (12)-(14) show the outcomes of the potential dimension. The coefficient is significantly positive, consistent with the previous results. Central China has the highest level of growth potential. The Results of model (15)-(17) indicate the outcome of the innovation competitiveness dimension. Central China and Eastern China show outcomes that are significantly positive, while Western China’s outcome is significantly negative. The results show that in Eastern and Central China, the impact of intelligent technology on industrial technology innovation is stronger than that in Western China, and Eastern China than Central China.

**Table 7:** The influence of the intelligentization of sub-region and sub-dimension on industrial competitiveness.

Variables	Bas			Cons			Pote			Inno		
	Eastern Region(6)	Central Region(7)	Westen Region(8)	Eastern Region(9)	Central Region(10)	Western Region(11)	Eastern Region(12)	Central Region(13)	Eastern Region(14)	Eastern Region(15)	Central Region(16)	Eastern Region(17)
*Int*	-0. 006** (-2. 16)	-0. 023*** (-4. 82)	-0. 031*** (-5. 39)	-0. 016*** (-6. 75)	-0. 011** (-2. 26)	-0. 025** (-. 23)	0. 008** (2. 26)	0. 051*** (6. 49)	0. 039** (2. 21)	0. 005** (2. 14)	0. 002*** (3. 35)	-0. 015*** (-3. 37)
*Ec*	0. 012*** (3. 86)	-0. 005 (-1. 03)	-0. 004 (-1. 00)	0. 020*** (7. 04)	0. 107*** (4. 15)	0. 011 (1. 46)	-0. 022*** (-6. 04)	0. 006 (1. 11)	-0. 006 (-1. 12)	0. 010** (2. 28)	-0. 001 (-1. 54)	-0. 004 (-1. 34)
*Tra*	-0. 001*** (-2. 93)	-0. 026*** (-5. 17)	0. 004 (1. 59)	-0. 002*** (-4. 61)	0. 003 (0. 51)	0. 001 (0. 16)	0. 002*** (4. 52)	0. 024*** (3. 68)	-0. 001 (-0. 19)	0. 001** (2. 04)	-0. 002** (-2. 22)	0. 010*** (4. 14)
*Es*	-0. 274*** (-5. 89)	-0. 126** (-1. 96)	-0. 090** (-2. 30)	0. 663*** (0. 067)	0. 153* (1. 82)	-0. 007 (-0. 08)	-0. 171*** (-3. 24)	-0. 184*** (-2. 67)	-0. 134* (-1. 66)	-0. 276*** (-5. 05)	-0. 023 (-1. 55)	0. 003 (0. 10)
*Hc*	-0. 258 (-8. 57)	-0. 231*** (-5. 62)	-0. 155*** (-5. 16)	0. 107*** (5. 95)	0. 050 (1. 25)	0. 120*** (3. 07)	0. 343*** (9. 44)	0. 303*** (6. 47)	0. 113** (2. 56)	-0. 245*** (-6. 74)	0. 017*** (3. 36)	-0. 165*** (-4. 97)
*Ur*	0. 035*** (1. 24)	0. 095*** (-2. 87)	-0. 267 (1. 45)	-0. 112*** (-2. 60)	-0. 045 (-1. 23)	-0. 103 (-0. 76)	0. 013 (0. 43)	-0. 131*** (-2. 79)	0. 282*** (2. 81)	0. 136*** (4. 21)	0. 016** (2. 07)	-0. 151** (-2. 50)
*Constant*	1. 958(42. 42)	1. 949*** (39. 19)	1. 873*** (55. 05)	1. 304*** (32. 90)	1. 457*** (20. 23)	1. 457*** (20. 23)	1. 108*** (20. 85)	1. 403*** (29. 73)	1. 403*** (29. 73)	1. 752*** (38. 07)	1. 322*** (177. 95)	1. 733*** (38. 73)
*Wald*	497. 12***	617. 95***	377. 01***	440. 87***	28. 60**	28. 60**	210. 47***	54. 52***	54. 52***	111. 49***	8842394***	117. 88***
*Fixed effects*	Yes	Yes	Yes	Yes	Yes	Yes	Yes	Yes	Yes	Yes	Yes	Yes

The regression results in Tables 6 and 7 show great consistency, indicating that the results are robust. Here is a detailed analysis of the regression results. First of all, in 1997, China proposed the strategy that “Eastern China regions should actively implement industrial transfer to Central China and Western China regions”, and the economically developed Eastern China and Central China regions have been transferring some high-cost industries to Western China regions. Therefore, the industrial structures in Central China and Eastern China regions have been relatively stable. At the same time, intense environmental regulation will promote inter-regional industrial transfer and structural upgrading. Western China has a fragile ecological environment, meaning that the region is more environment-sensitive than Central China and Eastern China. Therefore, under the stimulation of technological progress, Western China is taking more noticeable industrial transfer. For instance, the regions with a higher economic level and more technological advantages, such as Sichuan Province and Chongqing Municipality, make further effort to transfer industrial production sectors to other provinces and cities in the further western regions. In other words, the technological innovation represented by intelligent technology is promoting the reshaping of industrial system in Western China. Secondly, there is a stronger positive relationship between the level of innovation competitiveness and the level of intelligentization in Eastern China. It indicates that economically developed regions do have stronger technological advantages. In addition, the coefficient of Western is negative, which demonstrates that the research and development level of intelligent technology in Western China is still weaker than that in Central China and Eastern China, and has not formed strong innovation competitiveness. Finally, the structure of the industrial economy in Eastern China is relatively stable in the whole economic system, and its growth potential is stronger, indicating that Eastern China is in the mature period of the industrial cycle. The above results also further verify hypothesis 1 and hypothesis 2, and show that the regional competitiveness of Chinese industry is affected by the level of intelligentization, and the transfer of industry from developed regions to undeveloped regions takes on different degrees.

In addition, endogeneity should be considered, which is mainly brought about by two elements: reverse causality and missing variables. All the regression above uses panel data, which avoids the problem of missing variables to a certain extent. In order to ensure the robustness of the results, the Instrumental Variable Method was used for regression on the basis of the above basic regression. In terms of the selection of instrumental variables, considering the close relationship between emerging technologies and the level of economic development, per capita GDP is selected as the instrumental variable, and the two-period lag item of *Int* is also added. In addition, drawing on the method of Nunn and Qian (2019) [120], this paper selects the proportion of the number of fixed telephone per ten thousand people in 1990 and the proportion of high-tech enterprises to industrial enterprises above designated size in 1999 to represent the level of early technology and communication convenience in each region. Considering that only one year’s data to be used as the instrumental variable will lead to immeasurability due to the application of the fixed model, the above two indicators are multiplied by the “investment in reconstruction and technical transformation projects” from 2002 to 2016 to form two interaction items, ending up with two new instrumental variables. The regression results are shown in Appendix 1, which reveal that all the instrumental variables adopted passed the over identification test of 90% significance, and the positive and negative of coefficients did not change significantly, with only few exceptions. In addition, Hausman test finds that OLS regression is more effective than the instrumental variable method, indicating that the core explanatory variables did not have obvious endogeneity problems. Therefore, the robustness of the above regression results is further vindicated.

### 4.2 Spatial regression results

The spatial Durbin regression for Model (2) is used. Before the regression, spatial correlation test should be carried out first, which is the premise for spatial regression. The test results of spatial correlation are shown in Appendix 2. According to the results, except for a few years, the index of intelligentization level has significant overall or local correlation, indicating that the intelligentization level of this region can affect the surrounding regions, and then the regression can be carried out. Similarly, according to the Hausmann test, fixed-effect regression was selected in this part.

Table 8 shows the regression results of economic weight matrix, Table 9 shows those of the Economic-Geographic Matrix. The basic competitiveness represented by models (34)-(37), (50)-(53), and the innovation competitiveness by models (46)-(49), (62)-(65) show significant spatial correlation. In the basic competitiveness dimension, the intelligent space coefficient is significantly positive, indicating a strong positive spatial effect. On the one hand, it is proved that the region with a higher intelligentization level has a positive spillover effect on the industrial production level of the neighboring region. On the other hand, the region with closer economic or geographical space has a more prominent positive effect. Specifically, Central China is stronger than Eastern China and Western China, higher than the national average level. In the innovation dimension, the spatial coefficient of intelligentization level is significantly negative, indicating that there is a negative spatial effect. Regions with higher levels of intelligentization experience crowding-out effect for the neighboring innovation capabilities, showing an innovation agglomeration. Such phenomenon is more prominent in Western China than in Central China, and the same in Central China than in Eastern China. It reveals that the innovation gap in Western China and Central China is gradually widening. The overall intelligentization level in the Eastern China is higher, and the technological innovation system in Eastern China is relatively stable. Furthermore, in the structural dimension, there is no obvious spatial effect in the whole country, or in Eastern China or Central China, but the spatial coefficient in Western China is significantly negative. The reason is that regions with similar economic levels are often geographically adjacent, which means fiercer regional competition, because these regions tend to adopt the same industrial development strategies; and from the perspective of industrial transfer, these regions tend to move similar traditional industries out and then develop emerging industries with greater technological dividends. In terms of potential competitiveness dimensions, regional data does not show any significant effects of space, except the national data. This indicates that in the potential competitiveness dimensions, intelligentization has a trans-regional spatial effect among Eastern China, Western China and Central China. The technological competitiveness advantage of the Eastern China developed regions will restrain the industrial economic growth potential of the undeveloped regions in the Central China and the Western China to some extent. In general, the results of spatial analysis further support Hypothesis 2, that is, industrial competitiveness transfer appears to be the absolute transfer of economic aggregate, but essentially it is the relative transfer following the comparative advantage theory. In the era of intelligentization-oriented development, developed regions not only have robust growth momentum and technology advantages, but to a certain extent, they also restrain the growth potential of the undeveloped regions in China.

**Table 8:** Analysis results of economic weight matrix.

Variables	*Bas*				*Cons*				*Pote*				*Inno*			
	Nationwide (34)	Eastern Region(35)	Central Region (36)	Western Region(37)	Nationwide (38)	Eastern Region(39)	Central Region (40)	Western Region(41)	Nationwide (42)	Eastern Region (43)	Central Region (44)	Western Region(45)	Nationwide (46)	Eastern Region(47)	Central Region (48)	Central Region (49)
*Int*	-0.021*** (-2.94)	-0.025*** (-3.44)	-0.037** (-2.32)	-0.047*** (-3.67)	-0.025*** (-4.60)	-0.032 (-5.97)	-0.018 (-1.37)	-0.056*** (-3.86)	0.031*** (3.14)	0.041*** (4.64)	0.039** (2.02)	0.136*** (4.29)	0.015*** (3.13)	0.016** (2.52)	0.015 (0.94)	-0.035* (-1.89)
[*WX*]*Int*	4.352*** (8.67)	3.06*** (4.66)	7.616** (2.36)	4.452** (2.06)	-0.061 (-0.16)	-0.758 (-1.04)	-0.580 (-0.31)	-3.663** (-1.75)	-2.626*** (-4.09)	-0.433 (-0.44)	-4.115 (-1.29)	3.894 (0.86)	-1.920*** (-5.84)	-2.303*** (-2.88)	-3.983*** (-2.60)	-5.074*** (-2.71)
*Control*	Yes	Yes	Yes	Yes	Yes	Yes	Yes	Yes	Yes	Yes	Yes	Yes	Yes	Yes	Yes	Yes
*Fixed effect*	Yes	Yes	Yes	Yes	Yes	Yes	Yes	Yes	Yes	Yes	Yes	Yes	Yes	Yes	Yes	Yes
*R* ^*2*^	0.4241	0.5403	0.5520	0.5060	0.5112	0.1778	0.2202	0.3327	0.1214	0.1441	0.1783	0.1793	0.2266	0.2002	0.1800	0.2073
*Hausman*	1478.91***	22.75***	18.74***	40.29***	39.44***	17.12***	-67.93	-18.55	12.99*	11.08*	2902.43***	26.26***	50.33***	28.66***	-83.81	14.46**
*Wald*	75.10***	21.71***	5.56**	4.24**	0.03	1.08	0.10	3.05*	16.71***	0.19	1.68	0.73	34.15***	8.31***	6.77***	7.34***
*LogL*	941.0229	928.1207	928.1207	928.1207	892.2938	928.1207	928.1207	928.1207	577.6625	928.1207	928.1207	928.1207	852.7254	928.1207	928.1207	928.1207

**Table 9:** Analysis results of economic-geographic weight matrix.

Variables	*Bas*				*Cons*				*Pote*				*Inno*			
	Nationwide (50)	Eastern Region(51)	Central Region (52)	Western Region(53)	Nationwide (54)	Eastern Region(55)	Central Region(56)	Western Region(57)	Nationwide(58)	Eastern Region (59)	Central Region (60)	Western Region(61)	Nationwide (62)	Eastern Region(63)	Central Region(64)	Central Region (65)
*Int*	-0. 021*** (-2. 93)	-0. 025*** (-3. 41)	-0. 037*** (-2. 32)	-0. 047*** (-3. 68)	-0. 025*** (-4. 59)	-0. 032*** (-5. 92)	-0. 018 (-1. 38)	-0. 056*** (-3. 85)	0. 030*** (3. 14)	0. 040*** (4. 58)	0. 039** (2. 02)	0. 1358*** (4. 29)	0. 148*** (3. 13)	0. 016** (2. 54)	0. 015 (0. 94) (0. 94)	-0. 035*** (-1. 88)
[*WX*]*Int*	8. 681*** (8. 59)	6. 216*** (4. 86)	15. 231*** (2. 38)	8. 923** (2. 07)	-0. 136 (-0. 18)	-1. 518 (-1. 05)	-1. 142 (-0. 31)	-7. 294*(-1. 75)	-5. 221*** (-4. 06)	-1. 029 (-0. 54)	-8. 308 (-1. 32)	7. 727 (0. 85)	-3. 829*** (-5. 84)	-4. 523*** (-2. 82)	-7. 887*** (-2. 60)	-10. 138*** (-2. 72)
*Control*	Yes	Yes	Yes	Yes	Yes	Yes	Yes	Yes	Yes	Yes	Yes	Yes	Yes	Yes	Yes	Yes
*Fixed effect*	Yes	Yes	Yes	Yes	Yes	Yes	Yes	Yes	Yes	Yes	Yes	Yes	Yes	Yes	Yes	Yes
*R* ^*2*^	0. 4241	0. 5394	0. 5519	0. 5057	0. 1946	0. 1776	0. 2207	0. 3330	0. 1208	0. 1438	0. 1781	0. 1703	0. 2265	0. 2000	0. 1795	0. 2075
*Hausman*	1018.63***	26.71***	-18.03	-101.73	39.93***	17.46***	-48.91	-6.33	13.04*	11.86*	3373.92***	16.99***	52.47****	32.62***	-35.34	14.62**
*Wald*	73. 84***	23. 57***	5. 67**	4. 29**	0. 03	1. 11	0. 10	3. 05*	16. 51***	0. 29	1. 75	0. 72	34. 11***	7. 59**	6. 74**	7. 38***
*LogL*	943. 0745	339. 4796	335. 9268	260. 5836	894. 8333	305. 6888	319. 0929	238. 9994	580. 1773	184. 4891	222. 6452	150. 1591	855. 2214	291. 8403	307. 1943	228. 4570

## V. Conclusion and suggestions

Industry is the field where intelligent technologies are adopted earlier than other fields, and the effect of its intelligentization level on its competitiveness level is a valuable lesson for other fields in the future. By analyzing the effect of industrial intelligentization level on the overall competitiveness and the competitiveness of various dimensions and regions, the paper finds: 1) In general, the region with a higher level of intelligentization has a lower overall level of industrial competitiveness and a lower proportion of industry in the economic system, which is in compliance with the “Gradient transfer theory”, and proves the existence of industrial competitiveness transfer. However, this shift does not represent an absolute abandonment of industrial development, but it is for the relocation of local production sectors whose labor costs are higher, so that the region retains greater R&D advantages and have stronger growth momentum 2) From the view of different regions, first of all, the regions with a higher level of intelligentization have a lower competitiveness level of the production sector, indicating that the absolute transfer exists within all the regions. Besides, the incentive and promotion effect of intelligent development on innovation and R&D in the Eastern region is obviously stronger than that in the Central and Western regions. And the region with a higher level of intelligent development has greater potential of industrial economic growth. Moreover, the region with a higher intelligent level has a positive effect on the production activities in the regions with similar economy and geographical proximity, but a negative effect on the innovation activities. It indicates that intelligent technology promotes technology agglomeration. 3) Government behavior, financial development and marketization level can significantly improve the level of intelligentization. However, intelligentization guided by government behavior has an adverse effect on industrial competitiveness, which leads to the result that financial development level also has the same effect, while on the contrary, the market-guided intelligentization has a positive role in promoting the competitiveness.

So, it is obvious that if industrial intelligentization wants to promote the realization of a healthy growth of industrial competitiveness and a high-quality development of the national economy, an in-depth plan shall be made. Accordingly, this paper puts forward the following suggestions:

First, we should make a rational distribution of industries and encouraging a healthy competition. To promote the intelligent development of industry, on the one hand, industrial transfer shall be done in line with the local conditions, that is, the development of industries shall be planned out according to the actual regional situation. The enterprises with development conditions, potentials and customers should be given intelligentization upgrading priority so as to enhance their competitiveness, while those energy-intensive and high-polluting industrial enterprises with high cost of modification, small market space should be closed resolutely so as to avoid unnecessary reform expenses after mandatory saving. On the other hand, an overall plan of intelligentization industrial system development should be made. For a long time, there exists overlapping and vicious competition of industrial development among the regions. With the promotion of “new infrastructure”, all regions will usher in a boom of intelligent technology investment with provinces and cities issuing guidance and plans, leading to serious industrial overlap. In this regard, a benign competition mechanism should be established in the cooperation between regions to promote industrial complementarity.

Second, we should take a market-oriented and policy-guided industrial intelligentization development. In Chinese industrial sector, it has always been the policy to guide the production capacity and the function of market tends to be weak in this respect. The abnormal production capacity structure has long been restricting the healthy development of industry. In this regard, taking advantage of the intelligent technology revolution, we shall, firstly, give freedom to the market, loosen the restriction reasonably on the fields for non-state-owned intelligent manufacturing enterprises, fully mobilize the innovation efficiency of the market, and try to avoid “customized innovation” by government policies. Secondly, the government should make good use of its role as a “night watchman”. On the premise of maintaining the lifeline of the national economy, the government should strive to create a sound and stable market environment, maintain healthy competition and avoid being offside. At the same time, the government should make good effect to formulating relevant industrial policies. Both wild activity and inactivity of government should be stamped out. The point is, when the explosive growth of emerging technologies leads to rapid changes in the market structure, the government should play a vital role.

Thirdly, we should support innovation and encouraging entrepreneurship. The characteristics of high integration make industrial intelligentization an opportunity for more people to be engaged in it. Intelligent industry is a new industrial system highly integrated with agriculture and service industry, and the industrial boundary is gradually blurred. Therefore, taking advantage of the long-term effect of intelligentization to industrial growth, the government should encourage enterprises to adhere to innovation by issuing policies and incentive measures, so as to maintain the technological commanding heights. Meanwhile, the government should encourage more qualified individuals to start business drawing on the industrial intelligent technology tide and guide the integration of innovation and startup activities of other industries with the intelligent industry, so as to improve the overall efficiency.

## Supporting information

S1 Appendix Regression results of instrumental variable method. (DOCX)

S2 Appendix Spatial correlation test results. (DOCX)

## References

[1] Shuai J, Peng XJ, Zhao YJ, Wang YL, Xu W, et al. A dynamic evaluation on the international competitiveness of China’s rare earth products: An industrial chain and tech-innovation perspective. Resources Policy. 2022; 75:102444. 10.1016/j.resourpol.2021.102444

[2] Chen W, Su Z, Wang Y, Wang Q, Zhao GL. Do the rank difference of industrial development zones affect land use efficiency? A regional analysis in China. Socio-Economic Planning Sciences. 2022; 80: 101168. 10.1016/j.seps.2021.101168.

[3] Competitive Industrial Performance Report 2020, United Nations Industrial Development Organization (UNIDO).

[4] Industrial Development Report 2022. United Nations Industrial Development Organization (UNIDO).

[5] Zhuang L, Ye C. Changing imbalance: Spatial production of national high-tech industrial development zones in China (1988–2018). Land Use Policy. 2020; 94:104512. 10.1016/j.landusepol.2020.104512

[6] Lin BQ, Zhou YC. How does vertical fiscal imbalance affect the upgrading of industrial structure? Empirical evidence from China. Technological Forecasting and Social Change. 2021; 170:120886. 10.1016/j.techfore.2021.120886

[7] Jefferson GH, Rawski TG. Enterprise Reform in Chinese Industry. The Journal of Economic Perspectives. 1994; 8(2):47–70. 10.1257/jep.8.2.47

[8] Liu Z. An Analysis of the Correlation Between GDP and the Primary Industry, Secondary Industry and Tertiary Industry in China. Statistics and Application. 2020; 09:163–171. 10.12677/SA.2020.92018

[9] Baldwin JR, Gorecki P, Caves RE, et al. The dynamics of industrial competition: The dynamics of competition. 1995; 1–7. 10.1017/CBO9780511664700.002

[10] Smith KG, Grimm CM, Gannon MJ, et al. Organizational Information-Processing, Competitive Responses and Performance in the US Domestic Airline Industry. Academy of Management Journal. 2017; 34:60–85. 10.5465/256302

[11] Zhang J, Dan G. Review on the Research of Dynamic Competition Theory. Journal of Human Resource & Sustainability Studies. 2014; 02(4):246–251. 10.4236/jhrss.2014.24026

[12] Machado PS, Trigg AB. On absolute and comparative advantage in international trade: A Pasinetti pure labour approach. Structural Change and Economic Dynamics. 2021; 59:375–383. 10.1016/j.strueco.2021.09.005

[13] Ricardo D. On the Principles of Political Economy and Taxation[M]. 2008.

[14] Toeroek A, Toth J. Open characters of innovation management in the Hungarian wine industry[J]. Agricultural Economics (AGRICECON). 2013; 59(9):430–439. 10.17221/24/2013-AGRICECON

[15] Chen M. Competitor Analysis and Interim Rivalry: Toward a Theoretical Integration. Academy of Management Review. 1996; 21:100–134. 10.5465/amr.1996.9602161567

[16] Aghion P, Dewatripont M, Du L, et al. Industrial Policy and Competition. Cepr Discussion Papers. 2015; 37. 10.2139/ssrn.1811643

[17] Thurow LC. The competitive advantage of nations. Competitive Intelligence Review. 1991; 2(1). 10.1002/cir.3880020121

[18] Cooke P. Regional Innovation Systems: Competitive Regulation in the New Europe. Geoforum. 1992; 23(3): 365–382. 10.1016/0016-7185(92)90048-9

[19] Huovari J, Kangasharju A, Alanen A. Constructing an Index for Regional Competitiveness. Springer Berlin Heidelberg. 2002. 10.1007/978-3-540-24823-1_7

[20] Aiginger K, Firgo M. Regional competitiveness under new perspectives. Social Science Electronic Publishing. 2015; 26. 10.2139/ssrn.2685585

[21] Oral M, Reisman A. Measuring industrial competitiveness. Industrial Marketing Management. 1988; 17(3):263–272. 10.1016/0019-8501(88)90009-0

[22] Sirikrai SB, Tang JCS. Industrial competitiveness analysis: Using the analytic hierarchy process. Journal of High Technology Management Research. 2006; 17(1):71–83. 10.1016/j.hitech.2006.05.005

[23] Liu Z, Zeng S, Sun D, Tam CM. How Does Transport Infrastructure Shape Industrial Competitiveness? A Perspective from Industry Dynamics. IEEE Transactions on Engineering Management. 2020; 99:1–16. 10.1109/TEM.2020.2986801

[24] Thakur R, Angriawan A, Summey JH. Technological opinion leadership: The role of personal innovativeness, gadget love, and technological innovativeness. Journal of Business Research. 2016; 69(8):2764–2773. 10.1016/j.jbusres.2015.11.012

[25] Kim JH, Choi SH, Park IW, et al. Intelligence Technology for Robots That Think [Application Notes]. IEEE Computational Intelligence Magazine. 2013; 8(3):70–84. 10.1109/MCI.2013.2264573

[26] Mcshane M, English J, Nirenburg S. Knowledge Engineering in the Long Game of Artificial Intelligence: The Case of Speech Acts. 2022. 10.48550/arXiv.2202.01040

[27] Szladow AJ. Application of Artificial Intelligence Technology to Increase Productivity, Quality and Energy Efficiency in Heavy Industry. 1995.

[28] Hulten CR, Bennathan E, Srinivasan S. Infrastructure, Externalities, and Economic Development: A Study of the Indian Manufacturing Industry. Social Science Electronic Publishing. 2006; 20(2):291–308. 10.1093/wber/lhj007

[29] Acemoglu D, Restrepo P. Robots and Jobs: Evidence from US Labor Markets. NBER Working Papers. 2017. 10.2139/ssrn.2940245

[30] Aghion P, Jones BF, Jones CI. Artificial Intelligence and Economic Growth. NBER Chapters, 2018; 237–282. http://www.nber.org/chapters/c14015

[31] Acemoglu D, Restrepo P. The Wrong Kind of Ai? Artificial Intelligence and the Future of Labor Demand. NBER Working Papers. 2018. 10.3386/w25682

[32] Peres RS, Jia X, Lee J, et al. Industrial Artificial Intelligence in Industry 4.0—Systematic Review, Challenges and Outlook. IEEE Access. 2020; 8:220121–220139. 10.1109/ACCESS.2020.3042874

[33] State Council of the People’s Republic of China. New Generation of artificial intelligence development planning [R].2017-07-08.

[34] Makridakis S. The Forthcoming Artificial Intelligence (AI) Revolution: Its Impact on Society and Firms. Futures. 2017; 90:46–60. 10.1016/j.futures.2017.03.006

[35] Brynjolfsson E, Hitt LM. Beyond Computation: Information Technology, Organizational Transformation and Business Perfbrmance. Journal of Economic Perspectives. 2002; 14(4):23–48. https://www.aeaweb.org/articles?id=10.1257/jep.14.4.23

[36] Venturini F. Intelligent technologies and productivity spillovers: Evidence from the Fourth Industrial Revolution. 2022; 194:220–243. 10.1016/j.jebo.2021.12.018

[37] Acemoglu D, Gancia G, Zilibotti F. Competing Engines of Growth: Innovation and Standardization. Journal of Economic Theory. 2012; 147(2):570–601. 10.1016/j.jet.2010.09.001

[38] Yang T, Yi XL, Lu SW, et al. Intelligent Manufacturing for the Process Industry Driven by Industrial Artificial Intelligence. Engineering. 2021; 7(9):1224–1230. 10.1016/j.eng.2021.04.023

[39] Hashmi AW, Mali HS, et al. Artificial intelligence techniques for implementation of intelligent machining. Materials Today: Proceedings. 2021. 10.1016/j.matpr.2021.11.277

[40] Fan HC, Hu YC, Tang LX. Labor costs and the adoption of robots in China. Journal of Economic Behavior & Organization. 2020; 186:608–631. 10.1016/j.jebo.2020.11.024

[41] Thakur JS. Role of Artificial Intelligence & Expert System in: Business Competitiveness. Gian Jyoti E-Journal. 2012; 1(2): 1–9. http://www.gjimt.ac.in/web/wp-content/uploads/2017/10/N9

[42] Jeong BD, Mi SH. Analysis of Competitiveness According to the Current State of Artificial Intelligence Industry of Major Countries. The e-Business Studies. 2018; 19(5):215–229. 10.20462/TeBS.2018.10.19.5.215

[43] Mao S, Wang B, Tang Y, et al. Opportunities and Challenges of Artificial Intelligence for Green Manufacturing in the Process Industry. Engineering. 2019; 5(6):995–1002. 10.1016/j.eng.2019.08.013

[44] Gevel A. The Nexus between Artificial Intelligence and Economics. Tilburg University, Center for Economic Research. 2012. https://link.springer.com/book/10.1007/978-3-642-33648-5

[45] Dekle R. Robots and industrial labor: Evidence from Japan. Journal of the Japanese and International Economies. 2020; 58:101108. 10.1016/j.jjie.2020.101108

[46] Bommer R. Environmental Policy and Industrial Competitiveness: The Pollution-Haven Hypothesis Reconsidered. Review of International Economics. 1999; 7(2):342–355. 10.1111/1467-9396.00168

[47] Liu J, Liu L, Qian Y, et al. The effect of artificial intelligence on carbon intensity: Evidence from China’s industrial sector. Socio-Economic Planning Sciences. 2021; 5:101002. 10.1016/j.seps.2020.101002

[48] Lee HJ, Oh H. A Study on the Deduction and Diffusion of Promising Artificial Intelligence Technology for Sustainable Industrial Development. Sustainability. 2020; 12(14):5609. 10.3390/su12145609

[49] Abdulov R. Artificial Intelligence as an Important Factor of Sustainable and Crisis-Free Economic Growth. Procedia Computer Science. 2020; 169:468–472. 10.1016/j.procs.2020.02.223

[50] Lieberherr B. Elsa B. Kania: Battlefield Singularity: Artificial Intelligence, Military Revolution, and China’s Future Military Power. Washington D.C.: Center for New American Security, November 2017. SIRIUS–Zeitschrift für Strategische Analysen. 2019; 3(2):198–200. 10.1515/sirius-2019-2015

[51] Liang X, Yu J, Zheng D. Research on "Intelligence+" Innovation of Product and Industry Based on the Application of Artificial Intelligence Technology[M]. 2021. 10.1142/9789811238727_0058

[52] Qiu J. Research and development of artificial intelligence in China. National Science Review. 2016; 3(4):538–541. 10.1093/nsr/nww076

[53] Wang FY, Lu R, Zeng D. Artificial Intelligence in China. IEEE Intelligent Systems. 2007; 23(6):24–25. 10.1109/MIS.2008.99

[54] EVERGRANDE RESEARCH INSTITUTE, China’s New Generation of Artificial Intelligence Technology Industry Development Report [R].2019.

[55] Hine E, Floridi L. Artificial Intelligence with American Values and Chinese Characteristics: A Comparative Analysis of American and Chinese Governmental AI Policies. Social Science Electronic Publishing. 2022. 10.2139/ssrn.4006332

[56] Kania E B. Artificial Intelligence and Chinese Power. 2017.

[57] Wu F, Lu C, Zhu M, et al. Towards a new generation of artificial intelligence in China. Nature Machine Intelligence. 2020; 2(6):312–316. 10.1038/s42256-020-0183-4

[58] Zeng J. China’s Artificial Intelligence Innovation:A Top-Down National Command Approach?. Global Policy. 2021; 12(3):399–409. 10.1111/1758-5899.12914

[59] Roberts H, Cowls J, Morley J, et al. The Chinese approach to artificial intelligence: an analysis of policy, ethics, and regulation. AI & Society. 2021; 36:59–77. 10.1007/s00146-020-00992-2

[60] Ren T, Luo T, Li S, et al. Review on R&D task integrated management of intelligent manufacturing equipment. Neural Computing and Applications. 2022; 334:5813–5837. 10.1007/s00521-022-07023-9

[61] Chen J, Chen D, Yao A. Trade development between China and countries along the Belt and Road: A spatial econometric analysis based on trade competitiveness and complementarity[J]. Pacific Economic Review. 2020; 25(2):205–227. 10.1111/1468-0106.12329

[62] Ng K, Chen CH, Lee C, et al. A systematic literature review on intelligent automation: Aligning concepts from theory, practice, and future perspectives. Advanced Engineering Informatics. 2021; 47:101246. 10.1016/j.aei.2021.101246

[63] Robert E, Lucas JR. Trade and the Diffusion of the Industrial Revolution. American Economic Journal: Macroeconomics. 2009; 1(1):1–25. 10.1257/mac.1.1.1

[64] Witlox F. Towards a Relational View on Industrial Location Theory. Tijdschrift voor Economische en Sociale Geografie. 2002; 91(2):135–146. 10.1111/1467-9663.00101

[65] Weber A. Theory of the Location of Industries. Nature. 1960; 15(1):1. 10.1501/SBFder_0000000514

[66] Martin P, Rogers CA. Industrial Location and Public Infrastructure. Journal of International Economics. 1995; 39(3–4):335–351. 10.1016/0022-1996(95)01376-6

[67] Viladecans-Marsal E. Agglomeration Economies and Industrial Location: City-Level Evidence. Journal of Economic Geography. 2004; 4(5):565–582. 10.1093/jnlecg/lbh040

[68] Amiti M. New Trade Theories and Industrial Location in the EU: A Survey of Evidence. Oxford Review of Economic Policy. 1998; (2):45–53. 10.1093/oxrep/14.2.45

[69] Posner MV. International trade and technical change. Oxford Economic Papers. 1961; 13(3):323–341. 10.1093/oxfordjournals.oep.a040877

[70] Vernon R. International investment and international trade in the product cycle. The International Executive. 1966; 8(4):16–16. 10.1002/tie.5060080409

[71] Porter ME, Scott S. Measuring the ’Ideas’ Production Function: Evidence from International Patent Output[R]. Harvard Business School Working Paper. 2001. 10.3386/w7891

[72] Klepper S, Gort M. Time Paths in the Diffusion of Product Innovation. The Economic Journal. 1982; 92(367):630–653. 10.2307/2232554

[73] Klepper S. Entry, Exit, Growth, and Innovation Over the Product Life Cycle. American Economic Review. 1996; 86(3):562–583. 10.2307/3502659

[74] Klepper S. Industry Life Cycles. Industrial and Corporate Change. 1997; 6(1):145–181. 10.1093/icc/6.1.145

[75] Krugman P. Development, Geography, and Economic Theory[M]. 1995. 10.7551/mitpress/2389.001.0001

[76] Lewis WA. The Evolution of the International Economic Order[M]. 1978. 10.1515/9781400868513

[77] Akamatsu K. A Historical Pattern of Economic Growth in Developing Countries. The Developing Economies. 1962; 14:3–25. 10.1111/j.1746-1049.1962.tb01020.x

[78] Kojima K. Direct Foreign Investment: A Japanese Model of Multinational Business Operations[M]. 2010. 10.4324/9780203845660

[79] Lewis A. Economic Development with Unlimited Supplies of Labour. The Manchester school of economic and social studies. 1954; 22(2):139–191. 10.1111/j.1467-9957.1954.tb00021.x

[80] Deng Q. Relocation moveson——It’s time for China’s textile industry to transfer. China Textile. 2009; 7:5. https://kns.cnki.net/kcms/detail/detail.aspx?FileName=YGFT200907011&DbName=CJFQ2009

[81] Liu Y, Dong F. How Industrial Transfer Processes Impact on Haze Pollution in China: An Analysis from the Perspective of Spatial Effects. International Journal of Environmental Research and Public Health. 2019; 16(3). 10.3390/ijerph16030423 30717159 PMC6388366

[82] Wei HK. Evaluation of Regional Industrial Competitiveness in China. China Industrial Economy. 2002; 11:54–62. 10.19581/j.cnki.ciejournal.2002.11.007

[83] Rauch JE. Does History Matter Only When It Matters Little? The Case of City-Industry Location. Quarterly Journal of Economics. 1993; 108(3):843–867. 10.2307/2118410

[84] Hanson GH. Regional adjustment to trade liberalization. Regional Science and Urban Economics. 1998; 28 (4):419–444. 10.1016/S0166-0462(98)00006-4

[85] Feng GF, Liu ZY, Jiang WD. An analysis on the trends, features and causes of industrial transfer among China’s eastern, central and western regions. Modern Economic Science. 2010; 32(02):1–10+124. https://kns-cnki-net.ezproxy.lib.szu.edu.cn/kcms/detail/detail.aspx?FileName=DJKX201002000&DbName=CJFQ2010

[86] Han F, Yang L. How Does the Agglomeration of Producer Services Promote the Upgrading of Manufacturing Structure?:An Integrated Framework of Agglomeration Economies and Schumpeter’s Endogenous Growth Theory. Management world. 2020; 36(02):72–94+219. 10.19744/j.cnki.11-1235/f.2020.0022

[87] Kampen JK, Tobi H. Social Scientific metrology as the mediator between sociology and socionomy: a cri de coeur for the systemizing of social indicators. Social Indicators Stats Trends & Policy Development. 2011. https://library.wur.nl/WebQuery/wurpubs/418554

[88] Alain M, Quang V. Statistics and econometrics models. 1989. https://library.isical.ac.in/cgi-bin/koha/opac-detail.pl?biblionumber=10687

[89] Greenwood J, Seshadri A. Technological Progress and Economic Transformation. Economie d’Avant Garde Research Reports. 2002; 1:1225–1273. 10.1016/S1574-0684(05)01019-1

[90] Gourieroux C, Jasiak J. Financial Econometrics: Problems, Models, and Methods[M]. Princeton University Press, 2002. 10.1515/9780691187020

[91] Liu HN, Xiao H. Study on Pareto Improvement of Technological Progress, Economic Growth and Employment. 2017. 10.12783/dtssehs/mess2017/12130

[92] Lan S. Analysis on Technological Progress Contribution Rate to Economic Growth of Six Central China Provinces. 2007 International Conference on Wireless Communications, Networking and Mobile Computing. IEEE. 2007. 10.1109/WICOM.2007.1059

[93] Hui S, Fan Z, Ying Z, et al. Empirical research on competitiveness factors: Analysis of real estate industry of Beijing and Tianjin. Engineering. 2010; 17(3):240–251. 10.1108/09699981011038042

[94] Zhao P, Sun AY. Capital Structure and Industry Life Cycle:Empirical Research Based on Listed Companies of China. Journal of Industrial Engineering and Engineering Management. 2005. http://en.cnki.com.cn/Article_en/CJFDTOTAL-GLGU200503008.htm

[95] Ai HS, Yang XJ, Deng ZG. The effect estimation and channel testing of the technological progress on China’s regional environmental performance. Ecological indicators. 2015; 51:67–78. 10.1016/j.ecolind.2014.09.039

[96] Anselin L, Florax R, Rey SJ. Econometrics for Spatial Models: Recent Advances. Springer Berlin Heidelberg. 2004. 10.1007/978-3-662-05617-2_1

[97] Anselin L. Implicit functional relationships between systematic effects in a general model of movement. Regional Science & Urban Economics. 1982; 12(3):365–380. 10.1016/0166-0462(82)90024-2

[98] Anselin L, Bera AK, Florax R, et al. Simple diagnostic tests for spatial dependence. Regional Science & Urban Economics. 1993; 26(1):77–104. 10.1016/0166-0462(95)02111-6

[99] Burridge P. Testing for a common factor in a spatial autoregression model. Environment & Planning A. 1981; 13(7):795–800. 10.1068/a130795

[100] Anselin L. Spatial Econometrics: Methods and Models[M]. Springer Netherlands, 1988. https://link.springer.com/book/10.1007/978-94-015-7799-1

[101] Anselin L. The Maximum Likelihood Approach to Spatial Process Models. Springer Netherlands. 1988; 4:57–80. 10.1007/978-94-015-7799-1_6

[102] Lin J, Yu Z, Wei YD, et al. Internet Access, Spillover and Regional Development in China. Sustainability. 2017; 9(6):946. 10.3390/su9060946

[103] Li J, Wen J, Jiang B. Spatial Spillover Effects of Transport Infrastructure in Chinese New Silk Road Economic Belt. International Journal of e-Navigation and Maritime Economy. 2017; 6:1–8. 10.1016/j.enavi.2017.05.001

[104] Acemoglu D, Restrepo P. Robots and Jobs: Evidence from US Labor Market. NBER Working Papers. 2017. 10.2139/ssrn.2940245

[105] Autor D, Salomons A. Is Automation Labor-Displacing? Productivity Growth, Employment, and the Labor Share. NBER Working Papers. 2018. 10.3386/w24871

[106] Sun Z, Hou YL. How Does Industrial Intelligence Reshape the Employment Structure of Chinese Labor Force. China Industrial Economy. 2019; 05:61–79. https://kns.cnki.net/kcms/detail/11.3536.F.20190513.1354.008.html

[107] Liu L, Li L, Liu J, Cheng Z (2020) Intelligentization and the Transformation of Economic Development Mode: Theoretical Mechanism and Empirical Evidence. Economic Review. 2020; 02:3–19. https://kns.cnki.net/kcms/detail/11.3536.F.20190513.1354.008.html

[108] Dimmick J, Rothenbuhler E. The Theory of the Niche: Quantifying Competition Among Media Industries. Journal of Communication. 1984; 34(1):103–119. 10.1111/j.1460-2466.1984.tb02988.x

[109] Baum JAC, Oliver C. Toward an Institutional Ecology of Organizational Founding. Academy of Management Journal. 2017; 39(5):1378–1427. 10.5465/257003

[110] Zhu CQ. the niche ecostate-ecorole theory and expansion hypothesis. Acta Ecologica Sinica. 1997; 17(3):324–332. https://europepmc.org/article/CBA/533280

[111] Niu KC, Chu CJ, Wang ZH. Dynamic niche: a new foundation for rebuilding theory of community ecology. Scientia Sinica(Vitae). 2022; 52(03):403–417. 10.1360/SSV-2021-0160

[112] Patrik N, Sandoval CP, Svensson EI. Ecological Niche Dimensionality and the Evolutionary Diversification of Stick Insects. PLOS ONE. 2008; 3(4):e1907. 10.1371/journal.pone.0001907 18382680 PMC2270911

[113] Chevin LM, Decorzent G, Lenormand T. Niche dimensionality and the genetics of ecological speciation. Evolution. 2014; 68(5):1244–1256. 10.1111/evo.12346 24410181

[114] Takola E, Schielzeth H. Hutchinson’s ecological niche for individuals. 2021. 10.32942/osf.io/zrcbg

[115] Chentukov Y, Omelchenko V, Zakharova O, et al. Assessing the impact of higher education competitiveness on the level of socio-economic development of a country. Problems and Perspectives in Management. 2021; 19(2):370–383. 10.21511/ppm.19(2).2021.30

[116] Sutton J. Quality, Trade and the Moving Windows: Competitiveness and the Globalization Process. The Economic Journal. 2007; 117(524):F469–F498. 10.1111/j.1468-0297.2007.02119.x

[117] Martins S, Veiga FJ. Government size, composition of public expenditure, and economic development. International Tax & Public Finance. 2014; 21(4):578–597. 10.1007/s10797-014-9313-4

[118] Li J. The estimation of China’s information industry capital stock by country and region: 1991~2008. 2010 2nd IEEE International Conference on Information and Financial Engineering. 2010. 10.1109/ICIFE.2010.5609271

[119] Wang X. Empirical Analysis of the Rationality of China’s Urbanization Level on National and Regional Levels. Journal of Urban Planning and Development. 2016; 143(2):04016035. 10.1061/(ASCE)UP.1943-5444.0000371

[120] Nunn N, Qian N. US Food Aid and Civil Conflict. American Economic Review. 2014; 104(6):1630–66. doi: 10.1257/aer.104.6.1630

