# Supplementary material for: Whether intelligentization promotes regional industrial competitiveness: Evidence from China
Source: PLoS One. 2022 Jul 27;17(7):e0271186. doi: 10.1371/journal.pone.0271186 (PMC9328515; doi:10.1371/journal.pone.0271186)
Supplement: S1 Appendix — (DOCX) [file pone.0271186.s001.docx]

**Appendix 1 Regression results of instrumental variable method**

| Variables | *Bas* | | | | *Cons* | | | | *Pote* | | | | *Inno* | | | |
| --- | --- | --- | --- | --- | --- | --- | --- | --- | --- | --- | --- | --- | --- | --- | --- | --- |
|  | [Nationwide](C:/Program%20Files%20(x86)/Youdao/Dict/8.9.6.0/resultui/html/index.html#/javascript:;)  （18） | Eastern Region（19） | Central Region  （20） | Western Region（21） | [Nationwide](C:/Program%20Files%20(x86)/Youdao/Dict/8.9.6.0/resultui/html/index.html#/javascript:;)  （22） | Eastern Region（23） | Central Region  （24） | Western Region（25） | [Nationwide](C:/Program%20Files%20(x86)/Youdao/Dict/8.9.6.0/resultui/html/index.html#/javascript:;)  （26） | Eastern Region（27） | Central Region  （28） | Western Region（29） | [Nationwide](C:/Program%20Files%20(x86)/Youdao/Dict/8.9.6.0/resultui/html/index.html#/javascript:;)  （30） | Eastern Region  （31） | Central Region  （32） | Central  Region  （33） |
| *Int* | -0. 034^***^  (-3. 20) | -0. 020*****  (-1. 66) | -0. 112^***^  (-3. 12) | -0. 071^*^  (-1. 94) | -0. 059^***^  (-4. 50) | -0. 060^***^  (-4. 06) | -0. 97^**^  (-2. 23) | -0. 071  (-1. 64) | 0. 064^**^  (2. 28) | 0. 053^*^  (1. 66) | 0. 209^**^  (2. 28) | 0. 233^**^  (2. 48) | 0. 030^**^  (2. 09) | 0. 028*  (1. 70) | 0. 001  (0. 02) | -0. 092^**^  (-. 188) |
| *Control* | Yes | Yes | Yes | Yes | Yes | Yes | Yes | Yes | Yes | Yes | Yes | Yes | Yes | Yes | Yes | Yes |
| [*fixed*](C:/Program%20Files%20(x86)/Youdao/Dict/8.9.6.0/resultui/html/index.html#/javascript:;) [*effect*](C:/Program%20Files%20(x86)/Youdao/Dict/8.9.6.0/resultui/html/index.html#/javascript:;) | Yes | Yes | Yes | Yes | Yes | Yes | Yes | Yes | Yes | Yes | Yes | Yes | Yes | Yes | Yes | Yes |
| *R^2^* | 0. 5337 | 0. 5594 | 0. 5012 | 0. 5531 | 0. 1658 | 0. 1696 | 0. 1217 | 0. 2346 | 0. 1226 | 0. 1364 | 0. 0872 | 0. 1636 | 0. 1652 | 0. 1909 | 0. 1527 | 0. 1906 |
| *Wald* | 649894^***^ | 261751^***^ | 190292^***^ | 186529^***^ | 540787^***^ | 208371^***^ | 159395^***^ | 162130^***^ | 113080^***^ | 43780^***^ | 33949^***^ | 32696^***^ | 360869^***^ | 140957^***^ | 111243^***^ | 103302^***^ |
| *Hausman* | 18. 08^**^ | 0. 23 | 7. 99 | 0. 50 | 0. 07 | 1. 92 | 0. 51 | 0. 16 | 2. 17 | 0. 20 | 3. 14 | 0. 62 | 5. 67 | 1. 30 | 0. 02 | 0. 44 |
| *Overidentification*  *test* | 4. 097 | 2. 116 | 2. 169 | 4. 547 | 5. 524 | 2. 462 | 1. 539 | 4. 767 | 4. 821 | 0. 647 | 2. 132 | 3. 313 | 0. 381 | 3. 857 | 1. 658 | 6. 781^*^ |
