# Supplementary material for: Whether intelligentization promotes regional industrial competitiveness: Evidence from China
Source: PLoS One. 2022 Jul 27;17(7):e0271186. doi: 10.1371/journal.pone.0271186 (PMC9328515; doi:10.1371/journal.pone.0271186)
Supplement: S2 Appendix — (DOCX) [file pone.0271186.s002.docx]

**Appendix 2** Spatial correlation test results

| Year | Moran’s I | Geary’s |
| --- | --- | --- |
| 2003 | 0.105 | 0.408^**^ |
| 2004 | 0.217^**^ | 0.273^***^ |
| 2005 | 0.141^*^ | 0.382^**^ |
| 2006 | 0.190^**^ | 0.310^**^ |
| 2007 | 0.199^**^ | 0.319^**^ |
| 2008 | 0.187^**^ | 0.317^**^ |
| 2009 | 0.069 | 0.600^*^ |
| 2010 | 0.206^**^ | 0.326^**^ |
| 2011 | 0.201^**^ | 0.340^**^ |
| 2012 | 0.199^**^ | 0.328^**^ |
| 2013 | 0.193^**^ | 0.356^**^ |
| 2014 | 0.196^**^ | 0.350^**^ |
| 2015 | 0.187^**^ | 0.382^**^ |
| 2016 | 0.167^*^ | 0.403^*^ |
| 2017 | 0.175^**^ | 0.394^*^ |
